# Supplementary figures and images for: Modulation of sensory perception by hydrogen peroxide enables Caenorhabditis elegans to find a niche that provides both food and protection from hydrogen peroxide
Source: PLoS Pathog. 2021 Dec 23;17(12):e1010112. doi: 10.1371/journal.ppat.1010112 (PMC8699984; doi:10.1371/journal.ppat.1010112)

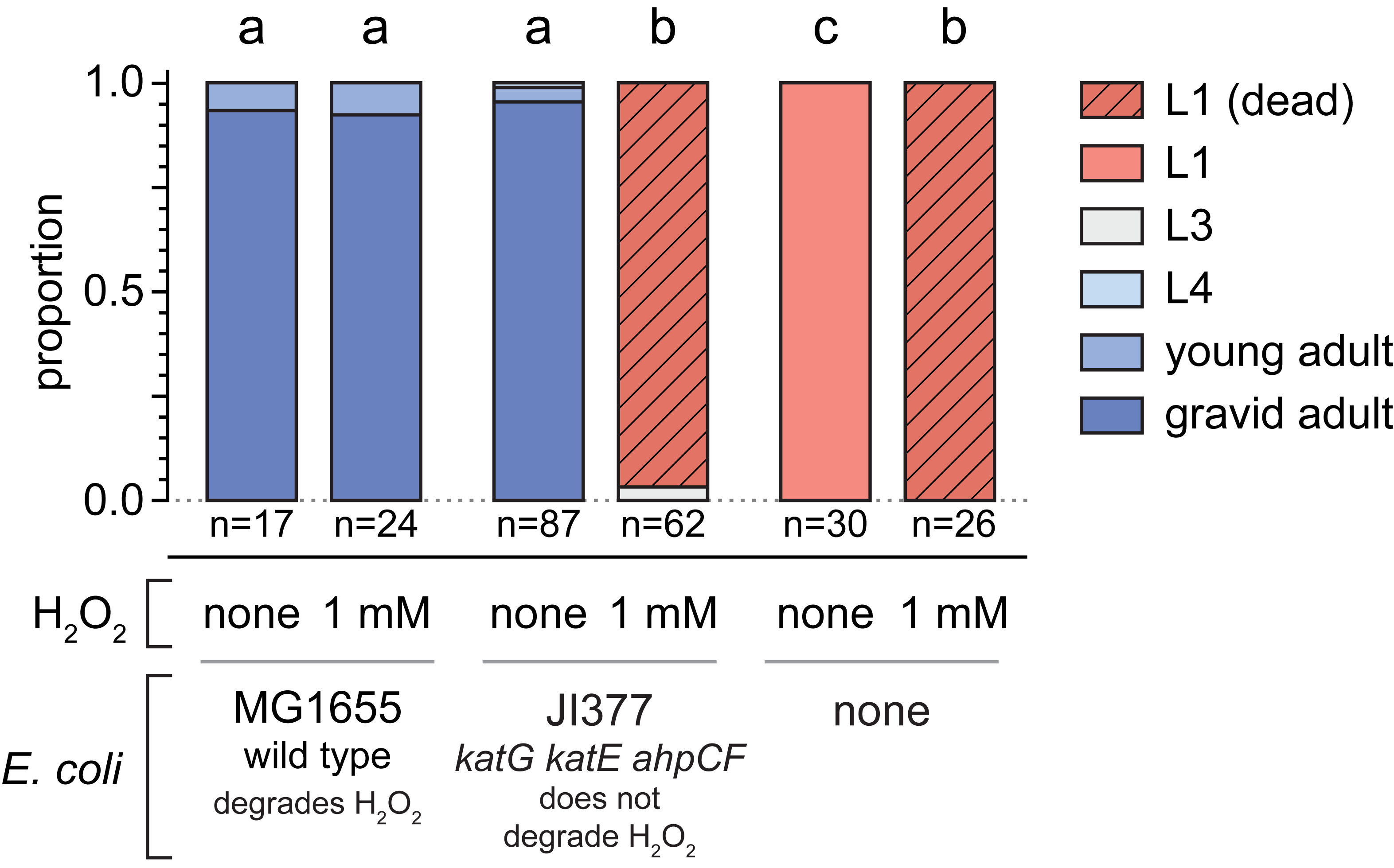

Supplement: S1 Fig — Development of wild-type C. elegans embryos in the presence of 1 mM H2O2. Groups labeled with different letters exhibited significant differences (P < 0.001, ordinal logistic regression) otherwise (P > 0.05). (TIF) [file ppat.1010112.s001.tif]

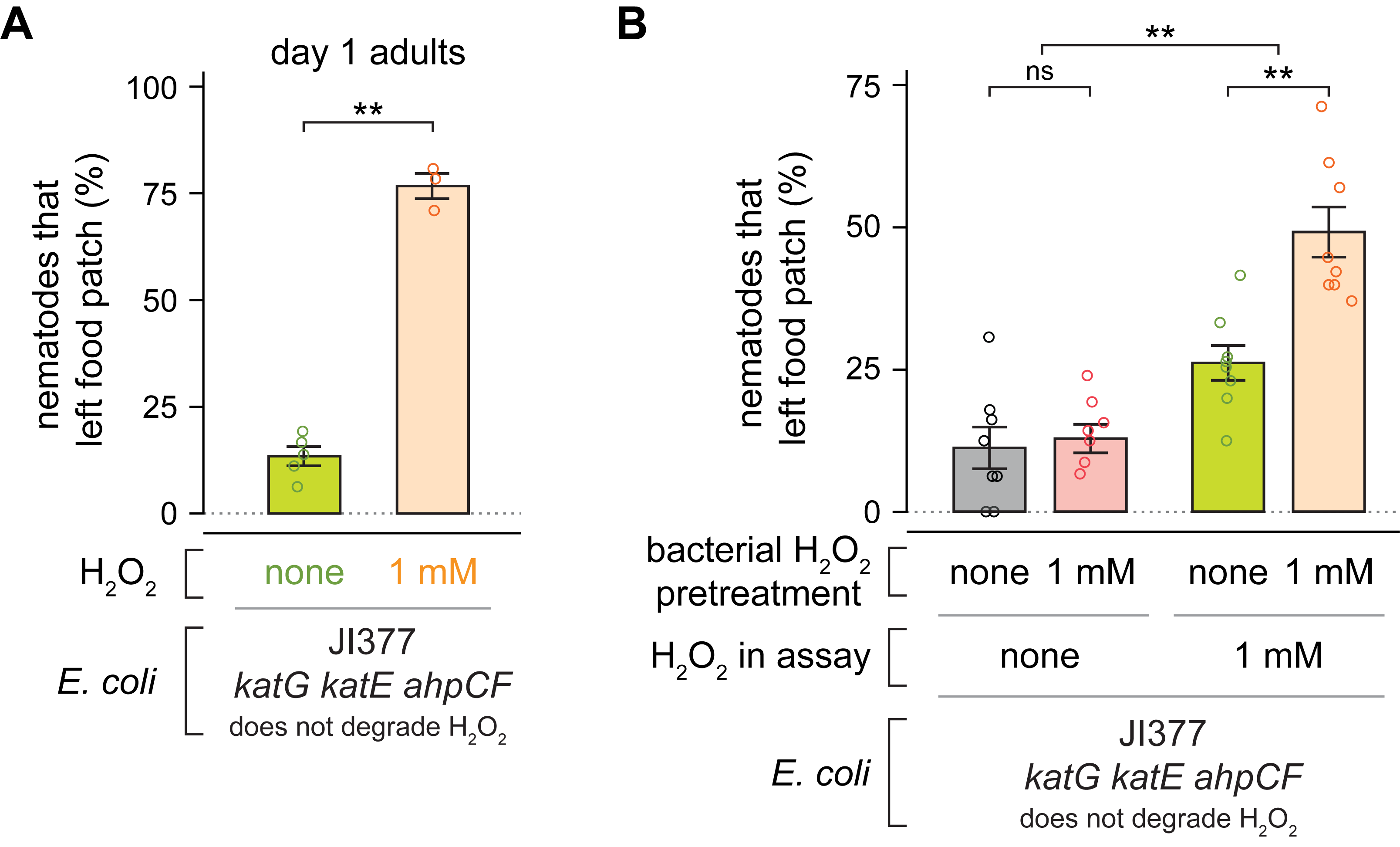

Supplement: S2 Fig — (A) H2O2 induced an increase in the proportion of day 1 adult C. elegans nematodes that left a patch of E. coli JI377. ** indicates P < 0.0001 (t-test). (B) Pre-treating the E. coli JI377 suspension used to make the lawn with 1 mM H2O2 for 20 hours did not increase nematode lawn leaving when no H2O2 was added to the assay plates, but caused a larger increase in lawn leaving when 1 mM H2O2 was added to the plates. ** indicates P < 0.006 and “ns” indicates P > 0.05 (standard least-squares regression). Data are represented as mean ± s.e.m. (TIF) [file ppat.1010112.s002.tif]

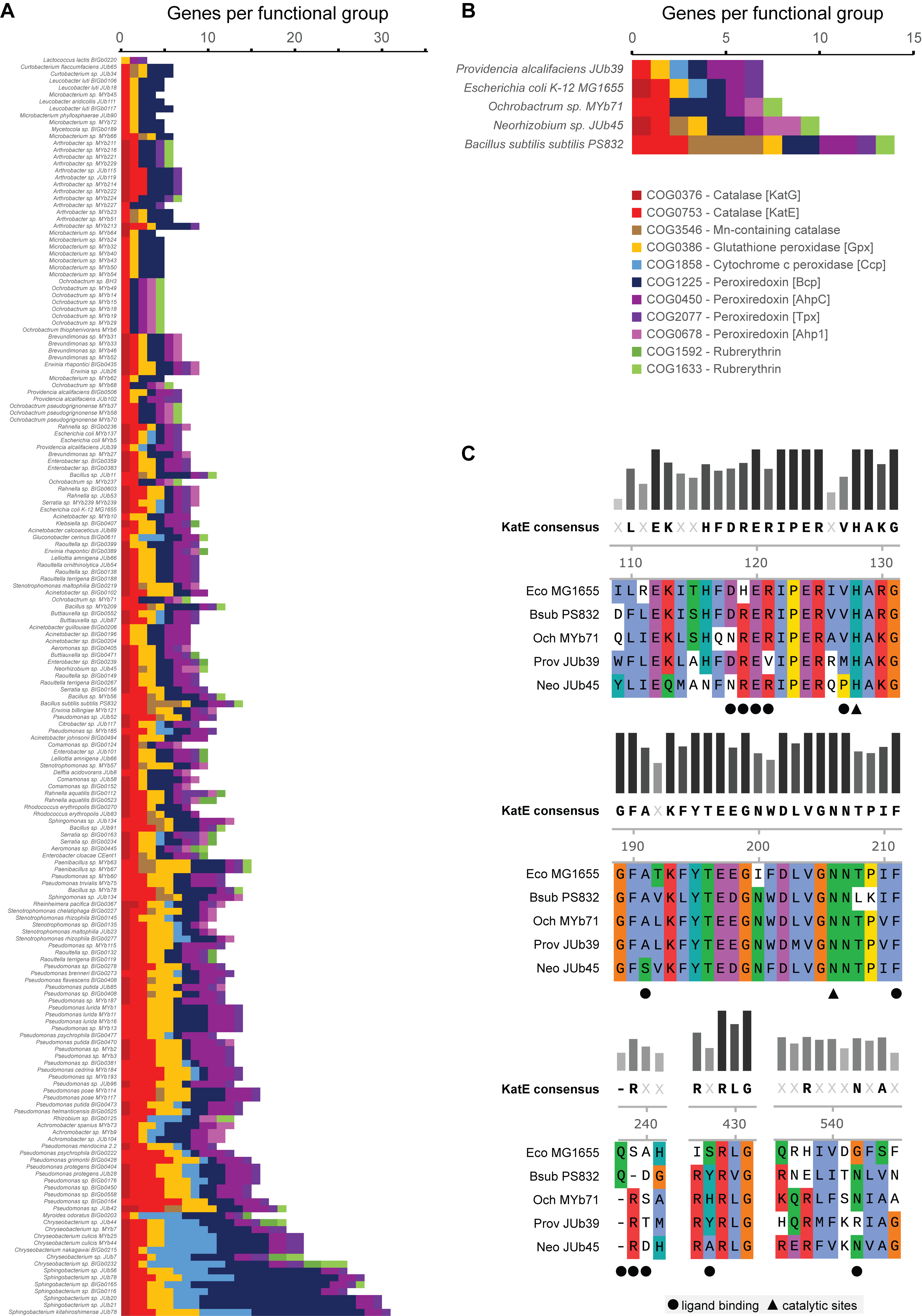

Supplement: S3 Fig — (A-B) We identified any genes within clusters of orthologous groups (COGs) associated with H2O2 degrading capabilities in 180 sequenced C. elegans microbiome genomes, plus E. coli MG1655 and B. subtilis PS832. These include catalase (COG0376, COG0753, COG3546), glutathione peroxidase (COG0386), cytochrome c peroxidase (COG1858), peroxiredoxins (COG1225, COG0450, COG2077, COG0678) and rubrerythrins (COG1592, COG1633). Total genes within each class are plotted for each strain. (C) Selected regions of protein alignments for a subset of the KatE orthologs (COG0753) are highlighted to show conservation of the catalytic residues (triangles) and variation in the predicted H2O2 ligand binding residues (based on E. coli). (TIF) [file ppat.1010112.s003.tif]

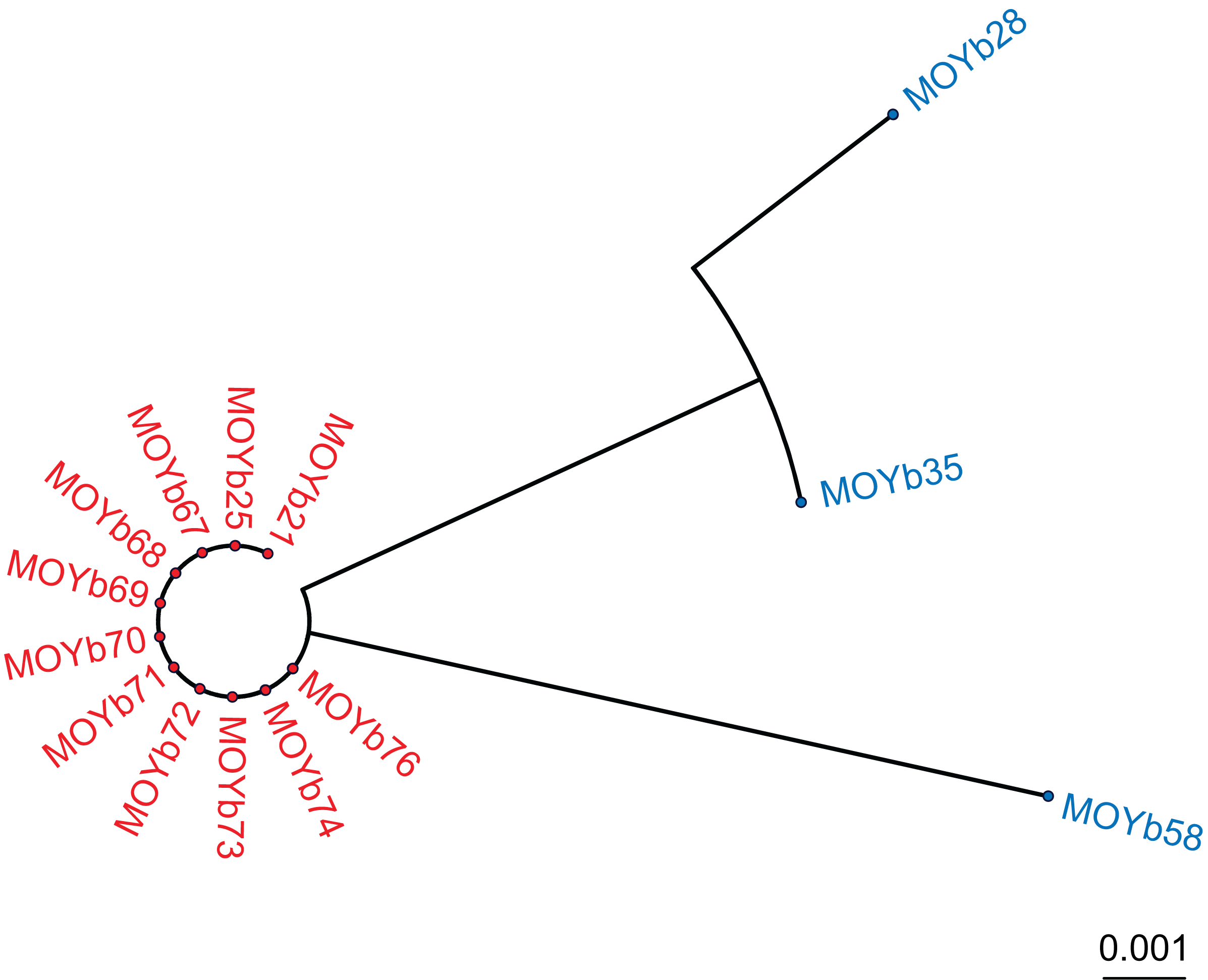

Supplement: S4 Fig — Phylogenetic tree, reconstructed using the neighbor-joining method based on partial 16S rRNA gene sequences, indicating the relationships among 14 Shewanella strains isolated from compost microbiome. The scale bar indicates the number of substitutions per site. Shewanella strains that supported C. elegans growth and reproduction are denoted in blue and strains that did not and were, instead, pathogenic to C. elegans are denoted in red. The isolation notes for these bacterial strains are shown in S5 Table. (TIF) [file ppat.1010112.s004.tif]

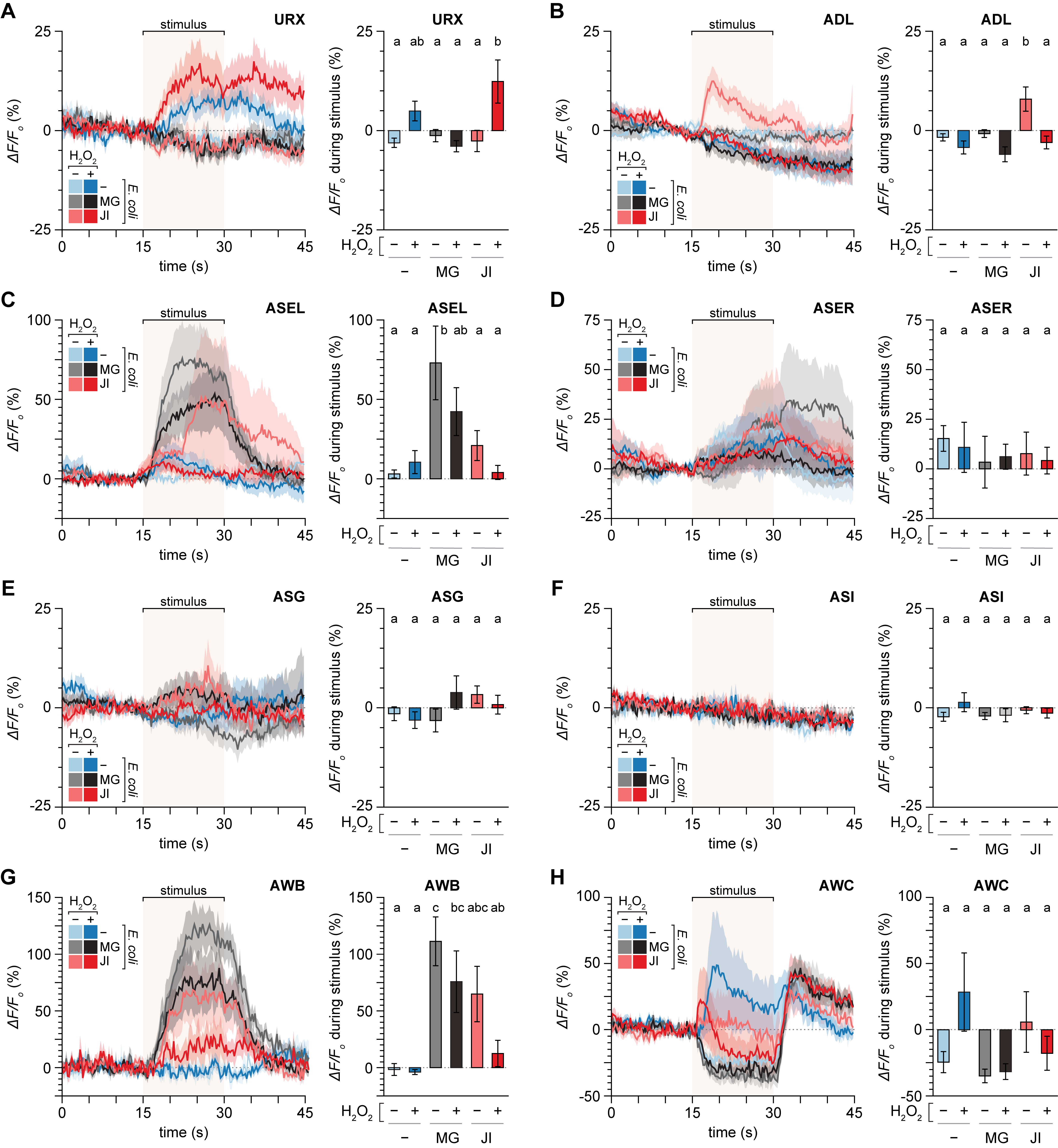

Supplement: S5 Fig — (A-H) Average GCaMP6 fluorescence traces of (A) URX, (B) ADL, (C) ASEL, (D) ASER, (E) ASG, (F) ASI, (G) AWB, and (H) AWC neuronal classes in response to six different stimuli (left sub-panels) and average changes in fluorescence in response to those stimuli (right sub-panels). The stimulus delivery interval is indicated by a shaded box. Data are represented as mean ± s.e.m. The number of neurons imaged was 28 ADF, 28 ADL, 14 ASEL, 14 ASER, 28 ASG, 28 ASH, 27 ASI, 28 ASJ, 28 ASK, 28 AWA, 13 AWB, 24 AWC, 18 BAG, and 28 URX. Groups labeled with different letters exhibited significant differences (P < 0.05, Tukey HSD test) otherwise (P > 0.05). Traces for the ASJ, ADF, AWA, BAG, ASK, and ASH neuronal classes are shown in Fig 4. (TIF) [file ppat.1010112.s005.tif]

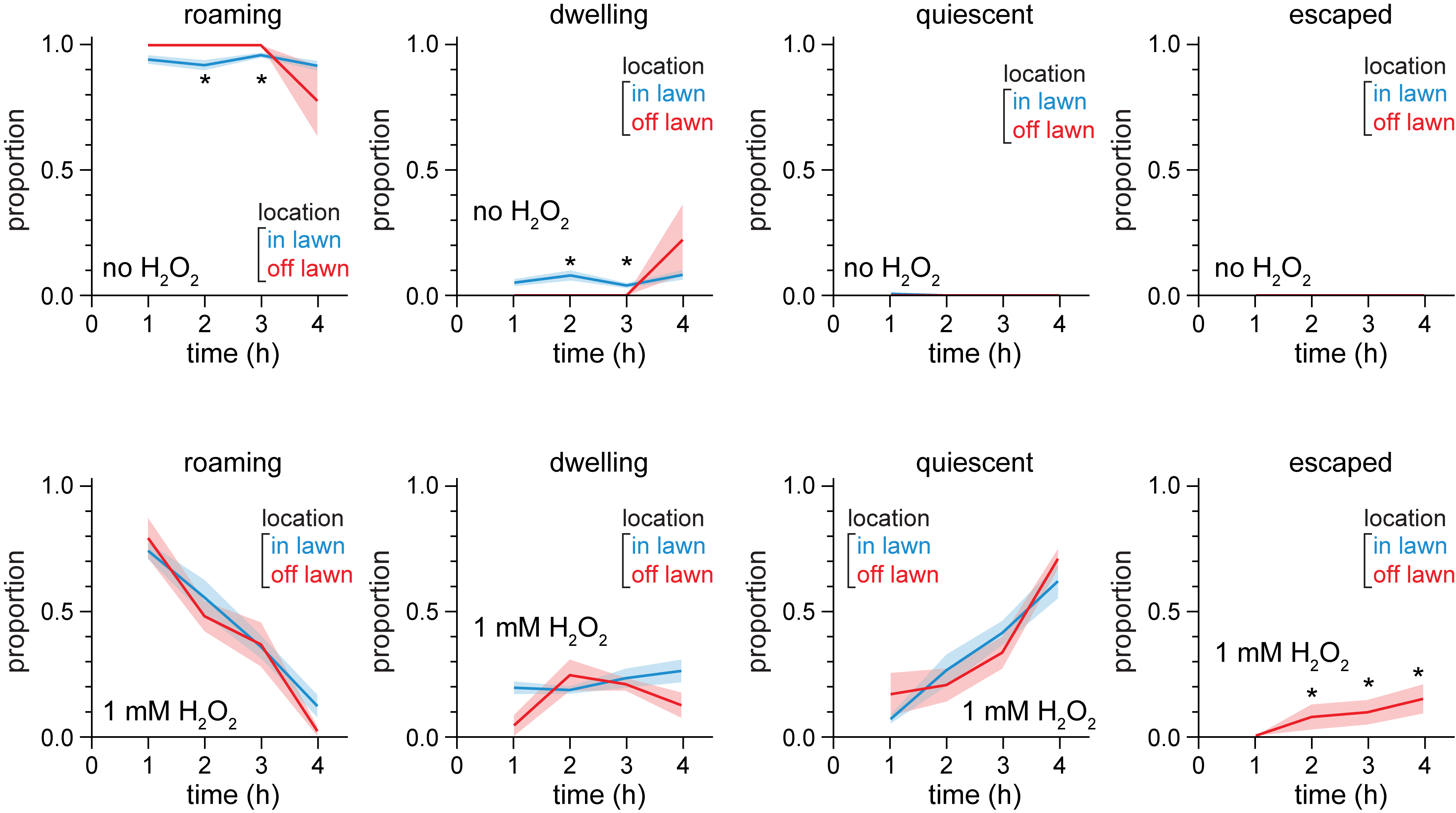

Supplement: S6 Fig — The plots show the proportion of animals roaming, dwelling, and quiescent that stayed in the E. coli JI377 lawn (blue) or left the lawn (red), and the proportion of animals that escaped the plate after leaving the lawn (red), in the assays on plates without H2O2 (top row) and on plates with 1 mM H2O2 (bottom row). Data are represented as mean ± s.e.m of the n = 6 assays per condition shown in Fig 6B. * indicates P < 0.05 otherwise P > 0.05 (t-test). (TIF) [file ppat.1010112.s006.tif]
